# Control of* Cryptococcus Gattii* Biofilms by an Ethanolic Extract of* Cochlospermum Regium* (Schrank) Pilger Leaves

**DOI:** 10.1155/2018/5764187

**Published:** 2018-06-06

**Authors:** Adriana A. Almeida-Apolonio, Wellinton J. Cupozak-Pinheiro, Vagner M. Berres, Fabiana G. S. Dantas, Terezinha I. E. Svidzinski, Kelly M. P. Oliveira, Marilene R. Chang

**Affiliations:** ^1^Faculdade de Medicina, Universidade Federal de Mato Grosso do Sul, Campo Grande, MS 79070-900, Brazil; ^2^Faculdade de Ciências Exatas e Tecnologia, Universidade Federal da Grande Dourados, Dourados, MS 79804-970, Brazil; ^3^Faculdade de Ciências Biológicas e Ambientais, Universidade Federal da Grande Dourados, Dourados, MS 79804-970, Brazil; ^4^Faculdade de Ciências da Saúde, Universidade Federal da Grande Dourados, Dourados, MS 79804-970, Brazil; ^5^Departamento de Análise Clínicas e Biomedicina, Universidade Estadual de Maringá, Maringá, PR 87020-900, Brazil

## Abstract

*Cryptococcus gattii* is an etiologic agent of cryptococcosis and a serious disease that affects immunocompromised and immunocompetent patients worldwide. The therapeutic arsenal used to treat cryptococcosis is limited to a few antifungal agents, and the ability of* C. gattii* to form biofilms may hinder treatment and decrease its susceptibility to antifungal agents. The objective of this study was to evaluate the antifungal and antibiofilm activities of an ethanolic extract of* Cochlospermum regium* (Schrank) Pilger leaves against* C. gattii*. The antifungal activity was assessed by measuring the minimum inhibitory concentration (MIC) using the broth microdilution technique and interaction of the extract with fluconazole was performed of checkerboard assay. The antibiofilm activity of the extract was evaluated in 96-well polystyrene microplates, and the biofilms were quantified by counting colony forming units. The extract showed antifungal activity at concentrations of 62.5 to 250 *μ*g/mL and when the extract was evaluated in combination with fluconazole,* C. gattii* was inhibited at sub-MIC levels. The antibiofilm activity of the extract against* C. gattii* was observed both during biofilm formation and on an already established biofilm. The results showed that the ethanolic extract of the leaves of* C. regium* shows promise for the development of antifungal drugs to treat cryptococcosis and to combat* C. gattii* biofilms.

## 1. Introduction

Cryptococcosis is a fungal infection that occurs worldwide. Despite the predominant result of opportunistic infections of immunocompromised patients, the incidence of infections among immunocompetent individuals is increasing [1–4]. Recently, studies have shown that cryptococcosis is a neglected tropical disease, although it is not recognized as such by the World Health Organization [5, 6]. Some factors that contributed to this conclusion included high mortality rates in treated patients (ranging from 20 to 60%) that can reach 100% in untreated patients [6].

Despite the importance of this disease, the treatment of cryptococcosis is limited to the antifungals fluconazole and amphotericin B, which are used alone or in combination with 5-flucytosine [7]. The primary etiological agents of cryptococcosis are species of the complexes* Cryptococcus neoformans* and* C. gattii* [8], which respond differently to the treatment established for meningoencephalitis [9].* C. gattii *is not only more clinically aggressive but also more difficult to control. Species of the* C. gattii* complex can compromise immunocompetent individuals and cause severe diseases of the central nervous system, such as meningitis, encephalitis, and meningoencephalitis [9]. In addition, lesions and long-term sequelae more commonly result from infections caused by species of the* C. gattii* complex than those caused by species of the* C. neoformans* complex [10]. Despite this disparity in symptoms, studies on possible new antifungals are primarily aimed at* C. neoformans* [11].

Species of the* C. gattii *complex have some attributes that can hinder antifungal therapy, such as heteroresistance to fluconazole [12]. In these cases, the treatment is limited to amphotericin B, which, although efficient, is highly toxic [13]. Another factor that can hinder antifungal therapies is the polysaccharide capsule, which, particularly for* C. gattii*, helps fungi escape the immune system [14] and decreases their susceptibility to antifungal agents [15]. In addition, the ability to form biofilms on host cells or medical devices, which is also promoted by the capsule, is a major factor associated with the high resistance of* Cryptococcus* spp. to antifungal drugs and host defense mechanisms [16].

In this context, it is of great importance to identify compounds with anti-*Cryptococcus *activity to aid in the treatment of cryptococcosis and alleviate the limitations of the current options. A promising alternative is the use of medicinal plants. Recently, Kumari et al. [17] evaluated the action of six essential oils extracted from medicinal plants on biofilms formed by* Cryptococcus *species, but these authors did not include isolates of* C. gattii*.

*Cochlospermum regium* (Schrank) Pilger, also known as yellow cotton tree, is a shrub found in the Brazilian cerrado, Paraguay, and Bolivia. It is a medicinal plant that is popularly indicated for the treatment of various diseases [18]. The extract of* C. regium *from roots presents antimicrobial action against* Staphylococcus aureus* and* Pseudomonas aeruginosa* and studies report the presence of various compounds such as phenolic, gallic acid, and ellagic acid [19–21], as well as the presence of sesquiterpenes in essential oil, organic acids, flavonoids, triterpenes, tannins, and steroids of leaves of* C. regium *[19, 22]. Recently, our research group showed antimicrobial and antibiofilm activity of a* C. regium* leaf extract against* Escherichia coli* and* Candida tropicalis* [22]. In this context, the aims of the present study were to evaluate the antifungal and antibiofilm activities of an ethanolic extract of* C. regium* leaves (Schrank) Pilger against* C. gattii*.

## 2. Materials and Methods

### 2.1. Plant Material

The leaves of* C. regium* (Schrank) P. were collected in the cerrado of Mato Grosso do Sul state (22°08'47.2”S; 054°54'54.1”W) under authorization number 57730-1 of the Biodiversity Authorization and Information System (SISBIO). The plant was identified in the herbarium of the Faculty of Biological and Environmental Sciences of the Federal University of Grande Dourados by Professor Dr. Zefa Valdivina Pereira. A sample of the species was deposited in the herbarium under DDMS registration 5001.

### 2.2. Production of a Crude Plant Extract

The leaves were dried in a circulating air oven at 30 °C and pulverized in a blade mill. The crude extract of the leaves of* C. regium* (ECR) was obtained by mixing 200 g of the powdered plant material with 1000 mL of 95% ethanol and maintaining the mixture at 25 °C for 72 h with shaking every 12 h. The obtained vegetal extract was rotoevaporated (Rotavapor R-215, Buchi) at 35 °C until complete volatilization of the solvent and then lyophilized using an EC MicroModulyo system coupled to a Savant VLP80 ValuPump vacuum pump.

### 2.3. *Cryptococcus gattii* Isolates and Growth Conditions

For the antifungal susceptibility and checkerboard assays, three* C. gattii *strains were assayed, including two isolated from sputum specimens (476 and 2164) and one from cerebrospinal fluid (CSF; 32G), obtained from the Microbiological Research Laboratory of the Federal University of Mato Grosso do Sul, Campo Grande, Mato Grosso do Sul, Brazil. A reference strain,* C. gattii *(ATCC 56990, Rockville, MD, USA), was included in all assays.

In each experiment, the yeasts were subcultured in Sabouraud Dextrose Agar (SDA; Sigma-Aldrich) for 48 h at 35 °C. For antifungal susceptibility and checkerboard assays, the density of yeast cells suspended in a saline solution (0.85%) was adjusted to a transmittance of 88% at 530 nm using a spectrophotometer. Next, 1:50 and 1:20 cell dilutions were made in RPMI-1640 medium (Roswell Park Memorial Institute, Sigma-Aldrich) containing L-glutamine, buffered with 0.165 M MOPS (3-(N-morpholino) propanesulfonic acid, Sigma-Aldrich) and supplemented with 2% glucose.

### 2.4. Antifungal Susceptibility Assay on Planktonic* C. gattii* Cells

The antifungal activity of ECR was evaluated using the microdilution technique in broth, according to the standards of the* Clinical and Laboratory Standards Institute* (CLSI, M27-A3) [23], with some modifications for natural products.

The ECR was dissolved in 80% dimethyl sulfoxide (DMSO, Sigma-Aldrich, USA) and serial dilutions of the extract were performed in 96-well flat-bottom microplates (Kasvi) containing RPMI-1640 medium. The ECR concentrations assayed were 1.95, 3.90, 7.81, 15.62, 31.25, 62.50, 125, 250, 500, and 1000 *μ*g/mL, and the microplates were incubated for 48-72 h at 35 °C.

Minimum inhibitory concentrations (MICs) were determined as the lowest ECR concentration that prevented visible growth of* C. gattii* after incubation. Fluconazole was used as a control, and the assay was performed in duplicate at two different times.

### 2.5. *In Vitro* “Checkerboard” Assay on Planktonic* C. gattii* Cells

An assay to evaluate the combined effect of ECR and fluconazole was performed based on the CLSI M27-A3 [23]. The final concentrations assayed were 3.9-500 *μ*g/mL for ECR and 0.5-64 *μ*g/mL for fluconazole. Together, the drugs formed a matrix of combinations of different concentrations in 96-well flat-bottom microplates, which were incubated for 48-72 h at 35 °C.

The interpretation of results was based on the fractional inhibitory concentration index (FICI), defined as follows: FICI=(ECR MIC in combination/ECR ECM alone)+(MIC of fluconazole in combination/MIC of fluconazole alone). The antifungal activity of the extract and the antifungal combination was interpreted as synergistic (FICI≤0.5), additive effect (0.5<FICI<1), indifferent (1≤FICI<4), or antagonist (FICI≥4) [24].

### 2.6. Evaluation of the Antibiofilm Activity of ECR

*Growth Conditions for C. gattii ATCC 56990. *For the ECR activity assays on biofilms,* C. gattii* ATCC 56990 was cultured in Sabouraud dextrose broth (HiMedia) for 24 h at 30 °C with shaking. The cells were collected by centrifugation at 5000 g for five min, washed three times with phosphate-buffered saline (PBS), and resuspended in RMPI-1640 medium. Using a Neubauer chamber, the cell density was adjusted to 1x10^8^ colony forming units (CFUs)/mL [25].

*Activity of ECR on Biofilm Formation by C. gattii ATCC 56990. *The effect of ECR on biofilm formation was assessed according to Martinez and Casadevall [25], with minor modifications. The assay was performed in 96-well polystyrene polystyrene microplates, with 2.5, 5, or 10 mg/mL of ECR added simultaneously to wells with the yeast. To form biofilms, the microplates were incubated at 35 °C for 48 h with shaking. A positive control (fungal cells and broth) and a negative control (broth only) were included.* C. gattii* biofilms were washed three times with 0.05% Tween 20 in trisphosphate buffer (TBS) to remove the nonadherent cryptococcal cells. The biofilms were characterized by the* C. gattii* viability assay to determine the CFUs, and the experiment was conducted in triplicate.

*Activity of ECR on Preformed Biofilms. *The effect of ECR on preformed biofilms was evaluated according to Martinez and Casadevall [25], with minor modifications. The assay was performed in 96-well bottom polystyrene microplates, with* C. gattii* ATCC 56990 added in RPMI-1640 broth. To form biofilms, the microplates were incubated at 35 °C for 48 h with shaking.* C. gattii* biofilms were carefully washed three times with 0.05% Tween 20 in TBS to remove the nonadherent cryptococcal cells. After washing, the biofilms were treated with 2.5, 5, or 10 mg/mL ECR. The microplates were incubated again at 35 °C for 48 h with shaking. Next, the biofilms were washed three times with 0.05% Tween 20 in TBS to remove the nonadherent cryptococcal cells. A positive control (fungal cells and broth) and a negative control (broth only) were included. The biofilms were characterized by the* C. gattii* viability assay to determine the CFUs, and the experiment was conducted in triplicate.

*Viability of C. gattii Cells in a Biofilm after Being Treated with ECR. *The viability of* C. gattii* strains was determined in each of the biofilm assays described above. After the biofilms were scraped from the wells of the microplates, the resulting cell suspensions were vigorously vortexed for 5 min to disaggregate the cells. Serial dilutions (in PBS) of each cell suspension were prepared and plated by the ASD drop plate technique [26]. Plates were incubated at 35 °C for 24-48 h. After incubation, the CFUs per unit area (Log_10_ CFU/cm^2^) of microtiter plate well were enumerated [27].

### 2.7. Statistical Analysis

The results were evaluated by ANOVA, and the means were compared by Tukey's posttest. A value of* p*<0.05 was considered significant for all evaluations. Statistical and graphical analyses were performed using GraphPad Prism^®^ 7.0 (GraphPad Software, San Diego, CA, USA).

## 3. Results

The MIC results for ECR against* C. gattii* ATCC 56990 and three clinical* C. gattii* isolates (two from sputum and one from CSF) are presented in Table 1. All strains were inhibited by ECR, with MICs ranging from 62.5 to 250 *μ*g/mL.

When ECR was evaluated in combination with fluconazole,* C. gattii* was inhibited at sub-MIC levels. However, according to the observed FICI values (1 to 1.25), the combination of the two antifungal drugs was not different from the drugs alone (Table 1).

**Figure 1:**
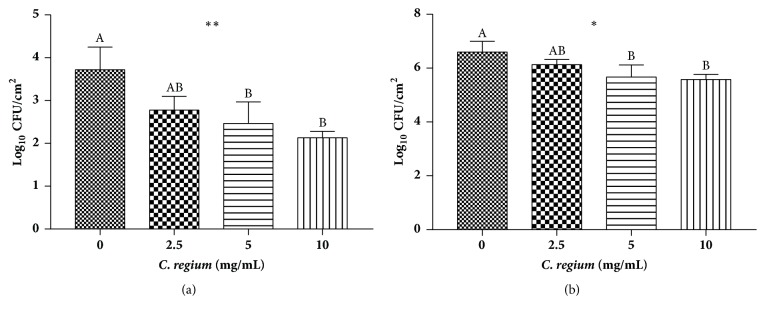
Activity of extract of* C. regium* against* Cryptococcus gattii* (ATCC 56990) biofilms. (a) Evaluation of ECR activity during biofilm formation by ANOVA and Tukey's posttest. *∗∗* (*p*<0.05) indicates a significant difference. Different letters over each column indicate a significant difference between samples (*p*<0.05). (b) Evaluation of ECR activity on preformed biofilms by ANOVA and Tukey's posttest. *∗* (*p*<0.05) indicates a significant difference. Different letters over each column indicate a significant difference between samples (*p*<0.05).

The activity of ECR on* C. gattii* ATCC 56990 biofilms is presented in Figure 1(a) (during biofilm formation) and Figure 1(b) (preformed biofilm) by determining the number of viable cells (in Log_10_ CFU/cm^2^). When comparing* C. gattii* ATCC 56990 biofilm formation in the presence and absence of ECR, the extract reduced the amount of biofilms formed by 25.33 to 42.58% in a dose-dependent manner, with a maximum observed reduction of 2 log_10_. For preformed biofilms, ECR reduced the amount of biofilms by 7.13 to 15.44% (Table 2).

## 4. Discussion

*C. gattii* is a primary etiological agent of cryptococcosis and is associated with severe and fatal cases of this disease. This encapsulated yeast can cause serious disease symptoms in humans and cause high levels of mortality. Unlike* C. neoformans*, which primarily infects immunocompromised patients,* C. gattii* is more often associated with infections of immunocompetent individuals [14].

In Brazil and in other countries where 5-flucytosine is not available, the treatment of* C. gattii*-induced cryptococcosis is limited to two drugs, fluconazole, and amphotericin B, which are frequently used in combination. However, this therapeutic choice has been questioned, since these two drugs may have variable interactions, from synergistic to antagonistic [28]. In addition, preventative measures for this disease are nonexistent, since it is caused by environmental yeasts that affect humans, primarily by inhalation. Thus, the transmission and virulence potential of this yeast combine to produce high mortality rates, justifying the search for new treatment or control options for this serious infection.

Among the attributes of* Cryptococcus* spp. that contribute to their virulence, the ability to form biofilms on abiotic surfaces is recognized as an important factor [29] and may help these environmental yeasts enter the human body. Thus, new therapeutic options aimed at preventing biofilm formation by cryptococcosis-causing fungi, such as* C. gattii*, would be useful in the prophylaxis of this disease but are currently unavailable, to the best of our knowledge. Unfortunately, neither fluconazole [25] nor amphotericin B [29] perform well on biofilms containing* Cryptococcus* spp.

The search for new compounds with anti-*Cryptococcus *properties is of great importance to maximize treatment options and prevent cryptococcosis. The results obtained in this study show that ECR could be a promising candidate to treat cryptococcosis caused by species of the* C. gattii* complex, since the extract presented MICs of 62 to 250 *μ*g/mL against the* C. gattii *species assayed, exhibiting good to moderate antifungal activity [30].

Considering our previous findings on the antimicrobial activity of ECR against other pathogenic microorganisms [22], we believe that this extract is even more promising for the treatment of hospital and domestic surfaces. This is because, considering its proven action against* E. coli*,* Candida tropicalis*, and now* Cryptococcus *(specifically the* C. gattii* complex of resistant species), ECR could be used to prevent biofilm formation. It is important to highlight that our previous study showed that ECR is innocuous for animal cells (92% cell viability for VERO lines) and did not show mutagenic potential in an Ames assay [22].

In the present study, we also evaluated the combination of ECR with fluconazole and observed that these antifungal drugs did not show a satisfactory interaction* in vitro*. Although this could be viewed as an unfavorable result, the concomitant use of both drugs in the same patient without one interfering with the antifungal activity of the other could be allowed. Thus, fluconazole could be used to treat cryptococcosis in parallel with ECR, avoiding the formation of biofilms on cell surfaces and preventing reinfections. Complementary studies could also validate the use of ECR as a coadjuvant in the treatment of cryptococcosis caused by* C. gattii *species.

The formation of biofilms contributes to the permanence of microorganisms in a host and helps protect against antimicrobials [31]. The yeasts of the genus* Cryptococcus* are endowed with a polysaccharide capsule composed primarily of glucuronoxylomannan, which is an important structural component of* Cryptococcus* biofilms [32].* Cryptococcus* spp. may form biofilms on polystyrene plates and medical devices [32, 33] and may be more resistant to amphotericin B and caspofungin [25]. The evaluation of viable cells is a direct method that allows quantifying the yeasts that present cell viability after treatment with a drug [27, 34]. The results of our study showed that ECR could reduce the biomass of a mature biofilm, according to the cell viability of the biofilm after treatment with the extract. However, ECR exhibited a superior antibiofilm activity when it was present during* C. gattii *biofilm formation, since an ECR concentration of 10 mg/mL resulted in a reduction of 42.58% of viable* C. gattii *cells compared to the control, indicating its potential use in prophylaxis.

## 5. Conclusions

The results of our study showed that an ethanolic extract of* C. regium* leaves showed antifungal activity and especially antibiofilm activity against* C. gattii*. Thus, the results of the present study expand the known antifungal properties of the leaves of this plant. However, additional tests are necessary to characterize the chemical compounds responsible for this antifungal activity. In addition, trials aimed at assessing the applicability of the extract at promoting positive health outcomes should be performed.

## Figures and Tables

**Table 1:** Antifungal activity of extract of *C. regium*, alone and in combination with fluconazole, against planktonic *Cryptococcus gattii* cells.

*Cryptococcus gattii*	Source	MIC ECR (*μ*g/mL)		MIC Fluconazole (*μ*g/mL)		FICI	Interpretation
		Alone	Combination	Alone	Combination		
32G	CSF	62.5	31.25	2	1	1	Indifferent
476	Sputum	125	31.25	8	8	1.25	Indifferent
2164	Sputum	125	62.5	2	1	1	Indifferent
ATCC 56990	Sputum	250	125	4	2	1	Indifferent

ECR: ethanolic extract from leaves of *Cochlospermum regium*; MIC: minimum inhibitory concentration; FICI: fractional inhibitory concentration index; CSF: cerebrospinal fluid.

**Table 2:** Effect of extract of the leaves of *C. regium* treatment on *Cryptococcus gattii* (ATCC 56990) biofilms.

ECR concentration (mg/mL)	During biofilm formation		Pre-formed biofilm	
	(Log_10_ CFU/cm^2^)	% reduction	(Log_10_ CFU/cm^2^)	% reduction
0	3.71	0	6.59	0
2	2.77	25.33	6.12	7.13
5	2.46	33.69	5.67	13.96
10	2.13	42.58	5.57	15.44

ECR: ethanolic extract from leaves of *Cochlospermum regium.*

## Data Availability

Previously reported cytotoxicity, mutagenicity, and antimicrobial activity data from* Cochlospermum regium* (Schrank) Pilger leaves ethanolic extract were used to support this study and are available at https://doi.org/10.1155/2017/4687154. This prior study is cited at relevant places within the text as [22].
